# Preliminary results, methodological considerations and recruitment difficulties of a randomised clinical trial comparing two treatment regimens for patients with headache and neck pain

**DOI:** 10.1186/1471-2474-10-115

**Published:** 2009-09-23

**Authors:** Willem De Hertogh, Peter Vaes, Dirk Devroey, Paul Louis, Hans Carpay, Steven Truijen, William Duquet, Rob Oostendorp

**Affiliations:** 1 Faculty of Physical Education and Physical Therapy, Vrije Universiteit Brussel, Brussels, Belgium; 2 Master in Manual Therapy Education, Faculty of Medicine and Pharmacy, Vrije Universiteit Brussel, Brussels, Belgium; 3 Department of Health Sciences, University College Antwerp, Antwerp, Belgium; 4 Department of General Practice, Vrije Universiteit Brussel, Brussels, Belgium; 5 Monica Hospitals, EFKA Campus, Department of Neurology, Antwerp, Belgium; 6 Tergooiziekenhuizen, Department of Neurology, Blaricum, The Netherlands; 7 Dutch Institute of Allied Health Care, Amersfoort, The Netherlands; 8 Scientific Institute for Quality of Healthcare, Centre for Allied Health Sciences, Radboud University Nijmegen Medical Centre, Nijmegen, The Netherlands

## Abstract

**Background:**

Headache is a highly prevalent disorder. Irrespective of the headache diagnosis it is often accompanied with neck pain and -stiffness. Due to this common combination of headache and neck pain, physical treatments of the cervical spine are often considered. The additional value of these treatments to standard medical care or usual care (UC) is insufficiently documented.

We therefore wanted to compare the treatment effects of UC alone and in combination with manual therapy (MT) in patients with a combination of headache and neck pain. UC consisted of a stepped treatment approach according to the Dutch General Practitioners Guideline for headache, the additional MT consisted of articular mobilisations and low load exercises.

Due to insufficient enrolment the study was terminated prematurely. We aim to report not only our preliminary clinical findings but also to discuss the encountered difficulties and to formulate recommendations for future research.

**Methods:**

A randomised clinical trial was conducted. Thirty-seven patients were included and randomly allocated to one of both treatment groups. The treatment period was 6 weeks, with follow-up measurements at weeks 7, 12 and 26. Primary outcome measures were global perceived effect (GPE) and the impact of the headache using the Headache Impact Test (HIT-6). Reduction in headache frequency, pain intensity, medication intake, absenteeism and the use of additional professional help were secondary outcome measures

**Results:**

Significant improvements on primary and secondary outcome measures were recorded in both treatment groups. No significant differences between both treatment groups were found. The number of recruited patients remained low despite various strategies.

**Conclusion:**

It appears that both treatment strategies can have equivalent positive influences on headache complaints. Additional studies with larger study populations are needed to draw firm conclusions. Recommendations to increase patient inflow in primary care trials, such as the use of an extended network of participating physicians and of clinical alert software applications, are discussed.

**Trial registration number:**

NCT00298142

## Background

Headache is a highly prevalent disorder with an important impact on society and on the individual sufferer [1]. Irrespective of the headache diagnosis it is often accompanied with neck pain and movement stiffness [2]. This combination of clinical signs occurs frequently in Tension-Type Headache (TTH), Migraine and Cervicogenic Headache (CEH), which are among the most prevalent headache types in primary care [3,4].

Patients with headache and neck pain or -stiffness are often referred for physical cervical spine treatment, such as manual therapy (MT) [5]. This consists mainly of specific joint mobilisations of the cervical spine in combination with exercise therapy to strengthen neck muscles and to improve active stability of the cervical motion segments. Various randomised clinical trials (RCTs) and systematic reviews demonstrate potential positive effects of this approach for different headache types [6-9].

Clinical guidelines for the medical/pharmaceutical treatment of headaches in primary care are available [10,11]. These guidelines provide diagnostic and therapeutic algorithms and stepped treatment plans for the most common headache types. The Dutch General Practitioners Guideline recommends a reserved attitude towards the prescription of physio- or manual therapy since the findings in literature are not conclusive [10]. The MIPCA guideline (Migraine In Primary Care Advisors) recommends physical therapy whenever a headache patient experiences neck stiffness [5,11]. The European Headache Federation suggest that physio- or manual therapy can be beneficial in some tension type headache patients, in this way giving some recommendation but not a firm one [4].

These diverse recommendations illustrate that the additional value of physical and manual therapy to usual care in patients with headache is insufficiently documented.

The aim of our study was to compare treatment effects of two primary care interventions, being the usual care (UC) and MT adjuvant to UC (UCMT).

## Methods

### Study design

We conducted a randomised clinical trial with blinded assessment and unblinded treatment, with a follow-up period of 52 weeks. We currently present the results of both treatment groups till the follow-up measurements of 26 weeks. The entire protocol is described in detail elsewhere [12] and was registered in clinicaltrials.gov (ClinicalTrials.gov identifier: NCT00298142). Approval for this study was obtained from the Medical Ethics Committees of the University Hospital of the Vrije Universiteit Brussel (UZ Brussel) and of the University Hospital of Antwerp (UZA).

We present a summary of the essential parts of the study design for the present article.

### Patients

#### Recruitment

Patients were recruited at general practitioners (GP's) offices, from outpatient clinics of the neurology department of the university hospitals UZ Brussel and UZA and via advertisements.

All participating medical doctors (GP's and neurologists) were contacted and informed personally about the study goals and protocol. Additional information was provided on an informative website [13] which included a link to an online eligibility screenings procedure, allowing easy referral of potential participants.

The advertisements (on leaflets and internet forums) contained a web link to a recruitment website [14]. On this site detailed information about the study was provided, followed by an online eligibility screenings procedure.

#### In- and exclusion criteria

Dutch speaking patients with a combination of recurrent headache and neck pain since minimum two months and at least twice a month with an active help-request (such headache burden that undergoing treatment is considered) were included. They had to be at least 18 years old and willing to participate.

Patients were excluded in case of cluster headache or trigeminal neuralgia, peripheral neuropathies and co-morbidity of chronic musculoskeletal disorders. Patients were also excluded in case of rheumatoid arthritis, Down syndrome and/or a history of surgery of the cervical region and in case of pregnancy. The presence of red flags for headache (warning signs for serious causes of the headache) was an extra exclusion criterion.

To avoid patients with treatment preferences, we excluded patients who received MT treatment for their headache during the last 12 months or patients whose prescribed medication was changed during the last two months.

The included headache types are migraine, TTH and CEH, as these are among the types of headache most frequently seen in general practice [4]. For migraine and TTH the criteria of the International Headache Society (IHS) were used [15], for CEH we used the criteria of the Cervicogenic Headache International Study Group (CHISG) [16].

For those patients responding to the advertisements, a Headache Impact Test (HIT-6, version with 6 questions) score of at least 56 points was required for inclusion, as from this critical point, the HIT-6 scoring advises the patient to contact his/her GP in order to start a treatment. All subjects signed a written informed consent prior to enrolment and prior to the baseline measurements. The informed consent was formulated according to the guidelines of the Medical Ethics Committee and contained, among others, specific information on the content of both treatment arms and the random allocation.

### Baseline measurements

Participants completed a diagnostic headache questionnaire [17]. Doing so, a systematic inventory is made of headache characteristics such as the localisation of the headache, pain intensity, frequency and duration of headache attacks. The completed questionnaires were retrospectively and independently screened by two raters (WDH and HC). Consequently a consensus diagnosis for each patient was made.

The Headache Impact Test (HIT-6) is used to measure the impact of the headache on the activities of daily living [18]. Headache related absenteeism and medical consumption (medication and health care contacts) of the last month are recorded retrospectively.

All questionnaires could be completed online or on paper.

### Randomisation and blinding

Randomisation was performed using blinded envelopes with a pre-stratification for the headache diagnosis. Pre-stratification was used to avoid the risk that patients of one headache type are allocated to one particular treatment group.

Subjects were randomly allocated to one of the two treatment groups after baseline measurements. The information of the completed questionnaires remained concealed ensuring blinded assessment.

Also for the follow-up measurements the rater remained blinded, as the online version was completed by the participating patients themselves, the paper versions were inserted in the computer software by an independent blinded rater.

### Follow-up measurements, primary and secondary outcome measures

Follow-up measurements were planned after 7, 12 and 26 weeks.

Global Perceived Effect (GPE) and the HIT-6 score were the primary outcome measures.

The GPE was measured using a 7 point scale, with scores ranging from 'completely recovered' to 'worse than ever' [18]. The score is dichotomised by labelling those subjects who perceive their headache complaints as 'completely recovered' or 'much improved' as responders. Those who rate their improvement as 'little improvement', 'no change, slightly worse, much worse and worse than ever' are labelled as non-responders.

Reduction in headache frequency, pain intensity, medication intake, absenteeism and headache related health care contacts were secondary outcome measures and were collected via a questionnaire.

### Interventions

The UCMT group received treatment during 6 weeks. It consisted of a combination of spinal mobilisations and low-load stabilising exercise therapy, following the protocol described by Jull et al[6]. A maximum of 12 sessions (twice a week over a period of 6 weeks) was provided. Each session lasted approximately 30 minutes.

To standardise MT treatment, all participants received a letter containing general recommendations for treatment, based on the available evidence [6,7], which was to be handed over to the therapist. Spinal mobilisations consist of low and/or high-velocity cervical joint mobilization techniques. Each therapist can decide which techniques of choice are selected for the treatment based on his own clinical skills and the patient's situation. Therapeutic exercises consist of low-load endurance exercises, more precisely cranio cervical flexion exercises.

To standardise UC, the protocol from the Dutch College of GPs was used as a guideline [10]. It consists of a stepped treatment approach. It has a part with patient education and treatment steps. In the treatment part, non-pharmaceutical as well as pharmaceutical interventions (prophylactic and attack medication) are described. The GP decides what therapeutic step needs to be taken based on the situation and history of the patient, e.g. by evaluating the effect of previously taken medication.

### Data reduction and analysis

All data were analyzed using the SPSS 12.0 for Windows. Normality was checked via the Kolmogorov-Smirnov test.

Differences between both treatment groups at baseline and follow-up measurements were analysed using Chi-square statistics for categorical variables. For continuous variables, comparisons between both groups baseline were made using an independent samples T-test. Comparisons between both groups at the follow-up measurements were made using a two-factor repeated measures ANOVA (group × time). Baseline values were included as the first follow-up measurements. Differences between successive measurements within a treatment group were analysed using a one factor repeated measures ANOVA. Significance value for all tests was set at p < .05.

Based on sample size calculations, a total of 93 subjects in each group was set as a target (significance level: p < 0.05, event rate in the UC group 0.50 and in the UCMT 0.70, power: 80% and an equal amount of subjects in both treatment groups).

The number of responders and non-responders was compared. The results of all subjects were analysed, regardless of their treatment adherence (intention-to-treat analysis). Differences of means and corresponding confidence intervals are displayed.

## Results

### Patient recruitment

**Figure 1:**
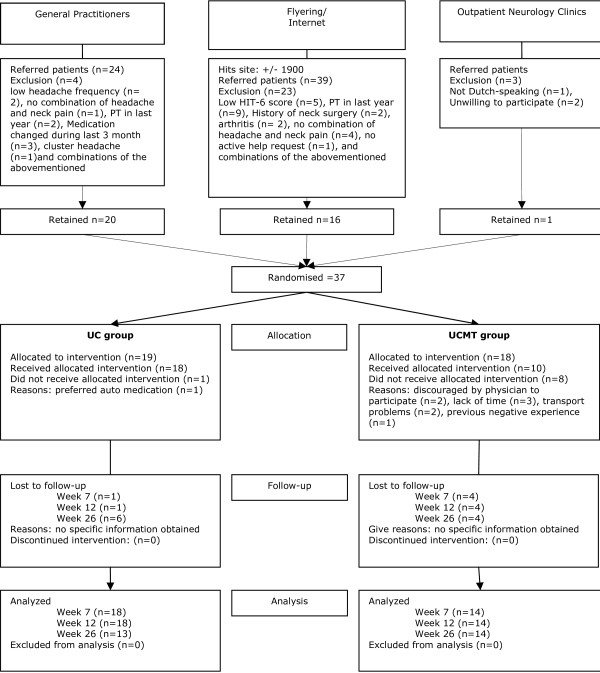
**Flow chart of subjects participation throughout trial**. PT: Physiotherapy; MT: Manual therapy, HIT: Headache Impact Test; UC: Usual Care; UCMT: Usual Care plus Manual Therapy.

Patients were recruited from February 2006 till June 2007. Figure 1 displays a flow chart of the study trajectory. In total 37 subjects were recruited. Retrospective analysis of the headache diagnosis questionnaire revealed 10 patients with Migraine, 23 with TTH, two with CEH and two with a combination of headaches.

### Baseline comparisons of both groups

Baseline headache characteristics of both groups are displayed in table 1. No significant differences were found between both groups.

**Table 1:** Baseline characteristics and differences between both treatment groups

	**UC (n = 19)**	**UCMT (n = 18)**	**Sign.**
Gender (M/F Ratio)	3/16	6/12	ns

Age (years, mean ± SD)	43.32 y ± 14.02 y	43.11 y ± 15.01 y	ns

Headache frequency (n)			
Daily	15	18	ns

Location Headache (n)			
Bilateral	8	6	ns
Bilateral dominant side	3	4	ns
Unilateral shifting	7	3	ns
Unilateral side locked	2	6	ns

Headache history			
> 1 year (n)	15	18	
Specification of headache duration (years; mean ± SD)	12.50 y ± 12.21 y	13.00 y ± 8.05 y	ns

Neck Pain Present (n)	18	16	ns

HIT-6 score (mean ± SD)	61.26 p ± 6.65 p	62.56 p ± 7.6 p	ns

Average Pain Intensity (VAS [mm]; mean ± SD)			
HA 4 weeks	67.64 ± 16.05	70.87 ± 20.16	ns
HA 3 months	66,26 ± 18,58	65.88 ± 18.38	ns
NP 3 months	45.67 ± 20.90	44.53 ± 20.23	ns
SP 3 months	19.11 ± 24.27	20.59 ± 20.07	ns

Numbers represent the proportion of the entire group which has a certain characteristic, unless specified otherwise. UC: Usual Care, UCMT: Usual Care plus Manual therapy, HIT-6: Headache Impact Test, 6 items (scores can range from 36-78 points), HA: Headache, NP: Neck Pain, SP: Shoulder Pain. VAS: Visual Analogue Scale (0 mm - 100 mm [0 = no pain; 100 = unbearable pain]), Sign.: Significant. ns: not significant (p > .05)

### Treatment content

**Figure 2:**
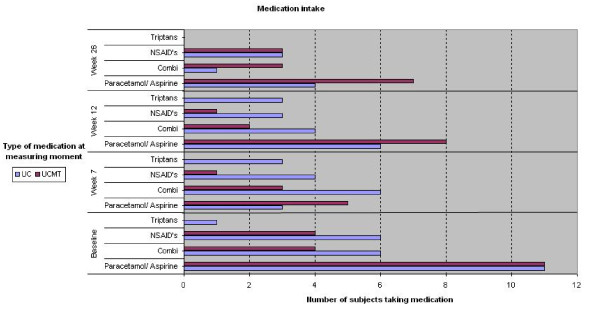
**Comparison of medication intake at baseline, week 7, week 12 and week 26**. No significant differences between both treatment groups were found (Chi-square statistics). Combi: Combined preparation, e.g. of paracetamol and caffeine, NSAIDs: Non Steroidal Anti-inflammatory Drugs. UC: Usual Care, UCMT: Usual Care plus Manual Therapy.

Medication intake of both treatment groups is graphically displayed in figure 2. Subjects from the UC group use more NSAIDs and triptans (both available on prescription). This difference was not statistically significant.

In table 2 the use of headache related health care contacts per provider is displayed. The GP and pharmacist are the most commonly visited health care providers.

**Table 2:** Overview of numbers of headache related health care contacts of both treatment groups

	**Week 7**		**Week 12**		**Week 26**	
	
	**UC** **(n = 18)**	**UCMT** **(n = 14)**	**UC** **(n = 18)**	**UCMT (n = 14)**	**UC** **(n = 13)**	**UCMT (n = 14)**
MT	1	10	2	7	1	/
GP	8	4	4	2	2	2
Neurologist	/	1	1	/	/	1
Pharmacist	6	4	5	2	6	/
Psychologist	1	2	/	2	/	/
Other	/	1	2	1	1	/

The number of people who contacted a health care provider is displayed. The total number of participants is displayed at the top of each column.

UC: Usual Care, UCMT: Usual Care plus Manual therapy. MT: Manual Therapy, GP: General Practitioner. The health care providers reported under the section 'other' are an osteopath, an orthomolecular doctor, a massage therapist (Shiatsu and Thai) and an internist.

In the UCMT group, 10/18 subjects actually visited a manual therapist after referral. The average number of visits was 8.6 at week 7. Between week 7 and 12, an average of 2 additional visits is reported.

### Primary outcome measures

The number of responders, derived from the GPE, is not significantly different between the two treatment groups. The HIT-6 score is significantly reduced at each point compared to baseline in both treatment groups. There were no significant differences between both treatment groups of follow-up measurements (table 3).

**Table 3:** Differences in primary outcome measures

**Variable**	**Treatment Group**	**Follow-up week 7**	**Follow-up week 12**	**Follow-up week 26**
Responders/Non responders (GPE)	UC	6/12	6/12	7/6
	UCMT	5/9	5/9	6/8
	Diff Proportion	3%	3%	11%

HIT-6 score (points)	UC	60.10 ± 5.55*	58.50 ± 4.62*	56.80 ± 6.46*
mean ± SD	UCMT	57.93 ± 4.58*	56.00 ± 6.95*	55.21 ± 9.75*
	Mean Diff	2.17	2.50	3.54
	CI	-2.12; 6.46	-2.74; 7.74	-5.76; 8.94

GPE: Global Perceived Effect; HIT-6: Headache Impact Test, 6 items (scores can range from 36-78 points), CI: Confidence interval; Diff: Difference between groups; UC: Usual Care; UCMT: Usual care plus Manual Therapy.

*: significant within group difference compared to value at baseline (Repeated Measures ANOVA, sign. level: p < .05)

### Secondary outcome measures

Headache intensity decreased in both treatment groups. Significant differences are flagged in table 4. Only within group differences were found.

**Table 4:** Differences in secondary outcome measures

**Variable**	**Treatment Group**	**Follow-up week 7**	**Follow-up week 12**	**Follow-up week 26**
VASHA3M (mm) Mean ± SD	UC	/	45.14 ± 25.71	38.43 ± 27.60
	UCMT	/	44.12 ± 24.15*	37.11 ± 26.52*
	Mean Diff	/	0.92	1.32
	CI	/	-25.92; 27.76	-28.27; 30.91

VASHA4W (mm) Mean ± SD	UC	40.78 ± 29.32*	36.78 ± 23.59*	32.11 ± 28.20*
	UCMT	40.73 ± 27.87*	28.00 ± 22.89*	33.18 ± 29.31*
	Mean Diff	0.05	8.78	-1.07
	CI	-26.88: 26.98	-13.14; 30.69	-28.29; 26.15

VASHANOW (mm) Mean ± SD	UC	31.91 ± 29.37	34.09 ± 28.19	13.55 ± 24.23
	UCMT	15.33 ± 24.33	15.25 ± 27.76	19.92 ± 29.09
	Mean Diff	16.57	18.84	-6.37
	CI	-6.73; 39.88	-5.44; 43.12	-29.71; 16.97

VASNP3M (mm) Mean ± SD	UC	/	24.29 ± 21.37*	24.43 ± 20.70*
	UCMT	/	28.67 ± 18.26	16.11 ± 18.56
	Mean Diff	/	-4.38	8.32
	CI	/	-25.63; 16.87	-12.77; 29.40

VASNPNOW (mm) Mean ± SD	UC	23.00 ± 29.18*	16.86 ± 22.39*	14.71 ± 20.23*
	UCMT	15.44 ± 22.02	18.56 ± 27.97	8.44 ± 15.36
	Mean Diff	7.66	-1.70	6.27
	CI	-19.83; 34.94	29.51; 26.11	-12.77; 25.31

50% Reduction HA Frequency. Number achieved/not achieved	UC	12/5	11/6	12/1
	UCMT	12/2	11/3	12/2
	Diff Prop	15%	14%	6%

Absenteeism (number absent/not absent)	UC	1/15	1/14	2/9
	UCMT	0/13	1/12	2/11
	Diff Prop	6%	1%	3%

VASHA3M: Average Headache Intensity of the last three months, VASHA4W Average Headache Intensity of the last four weeks, VASHANOW: Headache Intensity at the moment of testing

VASNP3M Average Neck Pain Intensity of the last three months, VASNPNOW Neck Pain Intensity at the moment of testing; Diff: difference between groups; Prop: proportion

HA: Headache. VAS: 100 mm Visual Analogue Scale (0 mm: no pain; 100 mm: unbearable pain)

*: significant within group difference compared to value at baseline (repeated measures ANOVA, sign. level: p < .05)

UC: Usual Care; UCMT: Usual Care plus Manual Therapy; Diff

The number of subjects where the headache frequency decreased with 50% is displayed in table 4. There were no significant differences between both groups.

## Discussion

We compared two treatment regimens for the treatment of patients with the combination of headache and neck pain in primary care. The two treatments being investigated were the GP treatment alone and in combination with manual therapy. The required number of patients could not be obtained within the timeframe that was foreseen for the entire project. Therefore the study was terminated prematurely.

The headache complaints evolved in both groups in positive sense such as a significant reductions in HIT-6 score and average headache intensity of the last four weeks. No significant differences between both treatment groups of follow-up measurements were found.

Due to the low number of recruited patients all our results should be interpreted cautiously. Despite this low number of participants, however, we report our results as the design of the study was published [12] and registered in a trial database [19]. By publishing the results of less successful trials, positive publication bias can be avoided and the results can be included in systematic reviews and meta-analysis. Moreover we propose to analyse causes of the limited number of included patients and formulate advice for further research in this domain in primary care.

### Study design

The study design was that of a pragmatic trial. In pragmatic trials the effectiveness of interventions is studied like they present in daily practice. The subjects can have a certain heterogeneity, as long as the spectrum of the population to which the studied treatments might be applied is represented [20,21]. The choice for a pragmatic design was based on two considerations. First, the observation in daily practice that patients with the combination of the clinical signs and symptoms headache and neck pain are frequently referred for physiotherapy or manual therapy treatment. An observation which is confirmed in literature [5,9,22]. Second, the description of beneficial effects of MT treatment for migraine, TTH and CEH [6,8,9,23]. This trial differs from previous trials because it investigates the additional value of MT as an adjuvant therapy to the usual care.

We included three headache types that are most frequently seen in primary care, and that are frequently accompanied with neck pain. This setup thus reflects daily practice, as patients from these three headache types can be exposed to the interventions studied. Taking these aspects in consideration, we believe the inclusion of more than one headache type is justified for our purposes. Unfortunately, the total number of participants remained far too low to allow subgroup analysis, with subgroups based on the headache diagnosis.

### Group characteristics

The total sample of patients studied here is representative for the entire headache population with regard to age and sex distribution [24,25]. The long history of headache complaints, the high frequency, pain intensity and high HIT-6 score indicate that the patients in our sample experienced a great burden of their headache.

Our participating patients were recruited in various ways (GP's offices, advertisements and outpatient neurology clinics). Recruitment via advertisements and outpatient neurology clinics was added to increase the number of participants [12]. Based on the exclusion and inclusion criteria and by means of a diagnostic questionnaire, complemented with the HIT-6, we have made an inventory of their headache characteristics and we presented a detailed description of their headache features. In all subjects a headache diagnosis was made based on responses to the questionnaire and using the revised IHS criteria for migraine and TTH [15] and the CHISG criteria for CEH [16]. Diagnoses were assessed independently, and in case of disagreement a consensus diagnosis was reached after discussing the case.

### Treatment effects

We were unable to demonstrate differences in treatment effects between both treatment groups at the follow-up measurements (week 7, 12 and 26). Both groups evolved positively, despite different accents in the treatment plan. E.g. the UC group received more medication on prescription. If confirmed in studies with larger patients samples this could indicate a potentially reduced risk for side effects of medication (e.g. gastro-intestinal bleedings) in the UCMT than in the UC group.

Both treatment regimens appear to be equivalent. Nonetheless, further research with larger patients samples is needed to clarify potential differences between both groups.

For future trials we recommended to use a headache diary. This can provide more complete and day by day information allowing a more profound recording of outcome measures.

### Treatment adherence and content

Of the original 18 patients in the UCMT group only 10 actually followed the suggested treatment. Of the remaining eight, five reported practical implications of MT treatment: lack of time to attend the therapeutic sessions regularly or transport difficulties. One patient was disappointed to be allocated in this treatment group, based on previous negative experiences with similar therapies. Two patients who entered the trial via the advertisements, were discouraged by their own physician to participate in the trial, without further specification of their arguments. We believe this is hard to avoid in the setup of a research protocol. As suggested by Vernon et al., maybe some kind of remuneration for participation could be useful to anticipate [26].

Based on the reports of the treating therapists, which described the treatment that was delivered, we have strong indications that the suggested treatment strategy in the UCMT group (a combination of spinal mobilisations and low load exercises) was actually followed. The average number of treatments was 8.6. This lies within the range of the number of treatments in studies using the same therapeutic approach [6,7]. No previous training period with the therapists was held due to practical reasons and to avoid withdrawal of therapists participation because of the time investment. For future research a training period with all participating therapists can be useful to standardise the delivered treatment even more.

Regarding the medication intake of patients, we see that the UC group received more medication for which a prescription is required, whereas in the beginning they mainly used over-the-counter medication. Subjects in this group receive more NSAIDS and triptans than the UCMT group and more than at baseline. This indicates that the stepwise pharmaceutical approach as described in the proposed guideline is followed by the GP's. The GP's who actually participated by referring patients were probably positively biased, knew guidelines and understood their potential in improving patient care. No side or adverse effects were reported by the participants, but more pro-active monitoring of potential adverse effects in either treatment arms is recommended for future trials.

### Patient enrolment

Sufficient patient enrolment is a key issue in every clinical trial. Recruitment difficulties are also reported by Vernon et al [26]. Apparently chronic headache patients are difficult to recruit in primary care settings and difficult to maintain within the boundaries of a treatment protocol. Our disappointing enrolment is probably the result of multiple factors.

Only half of the contacted physicians actually referred patients for the study, and most of them referred only one patient. Some of the documented reasons for insufficient participation of physicians are lack of time, difficulties with complex enrolment procedures, insufficient knowledge and awareness of the trial, concerns about the impact of trial participation on the doctor-patient relationship or about the loss of professional autonomy [27-29]. One recommends intrinsic motivation, frequent reminders and practically feasible study protocols to achieve a more successful participation of physicians.

We contacted all physicians personally (first by phone, followed by at least one visit). The research protocol was explained, the research question and its clinical relevance were discussed. All physicians recognised the frequently occurring and challenging combination of headache and neck pain. The clinical value of the research question was stressed as well as the importance of physician cooperation for clinical studies in primary care. These aspects are recommended to enhance the physicians motivation and to gain their commitment [29,30]. We provided additional study information on websites which contained also online enrolment procedures, making the referral procedure as easy as possible. Regularly reminder e-mails were sent to keep their attention with the study. Despite our efforts the number of referred and enrolled patients remained discouragingly low.

For future research we recommend the development of a far more extensive network of physicians, taking into account that only a limited number will actually participate and refer patients. For instance, Van der Wouden et al. found that only 30% of all contacted GP's actually participated in studies with an interventional setup [31].

The use of software applications which are incorporated in the patients electronic health record should be considered. Embi et al. developed and used a Clinical Trial Alert System which increased the physicians participation and patient enrolment significantly [28,32].

In our opinion, the fear of physicians to lose professional autonomy was a major issue. Clinicians have treatment preferences, and randomised study protocols require them to take a step back from daily routine, or even to prescribe a treatment they do not fully support. This can result in selective participation and referral of patients. Only those patients who do not fit in the regular schema or in who the regular schema was not successful are considered for referral to participate in the study.

Not only clinicians, but also patients have treatment preferences. Current legislation demands that patients are a priori informed in detail about the study purposes and procedures, by means of a written informed consent. Patients who prefer one of the treatment strategies in the study protocol are potentially biased or risk to be de-motivated when they are not allocated to the group they wanted to be allocated to. This might be one of the explanations for a number of not returned follow-up questionnaires and dropouts.

Treatment preferences of both physicians and patients can thus lead to insufficient enrolment and dropouts. The number of dropouts was equivalent in both treatment groups, so these factors have played equally in both groups.

As a remedy, the use of observational studies should be considered in future studies. E.g. Pfeiffer et al. used a prospective cohort study to avoid conflicts with physicians routine and treatment preferences of both physicians and patients [33].

If an observational study protocol results in a larger patient sample, the analysis of subgroups can be performed. The treatment that has been followed can be inserted in the analyses as independent variable.

Bell-Syer et al. recommend the use of wide inclusion criteria in the early screening of potential subjects [30]. To avoid biased patients we excluded patients who received MT treatment for their headaches during the last 12 months or patients whose prescribed medication was changed during the last two months. In this patient population these can be too strict exclusion criteria. In future research this exclusion criteria can be formulated less strict, which will probably result in a greater enrolment.

## Conclusion

The headache complaints of both treatment groups evolved positively. We were unable to demonstrate differences between both treatment groups. Future studies with larger patients samples are needed. To achieve a higher number of patients in future studies we recommend the use of an extensive network of participating physicians, the use of software applications incorporated in patients' electronic health record. The use of observational instead of randomised study protocols should be considered.

## Conflict of interests

The authors declare that they have no competing interests.

## Authors' contributions

WDH took part in all elements of the study and drafted the manuscript. PV, DD contributed to the design of the study, data interpretation and revision of the manuscript. PL, HC contributed to data interpretation and revision of the manuscript. ST contributed to the design of the study, statistical analysis, data interpretation and revision of the manuscript. WD contributed to the design of the study and statistical analyses. RO contributed to the design of the study, data interpretation and revision of the manuscript. All authors read and approved the final manuscript.

## Pre-publication history

The pre-publication history for this paper can be accessed here:


